# Cultural Adaptation of Digital Knowledge Translation Tools for Acute Otitis Media in Low- to Middle-Income Countries: Mixed Methods Usability Study

**DOI:** 10.2196/13908

**Published:** 2021-01-20

**Authors:** Salima Meherali, Lisa Hartling, Shannon D Scott

**Affiliations:** 1 Faculty of Nursing University of Alberta Edmonton, AB Canada; 2 Department of Pediatrics University of Alberta Edmonton, AB Canada

**Keywords:** acute otitis media, knowledge translation, pediatric, parent’s experiences, information needs, global health

## Abstract

**Background:**

Acute otitis media (AOM) is the most common pediatric bacterial ear infection. AOM presents challenges to parents who lack accurate information. Digital knowledge translation tools offer a promising approach to communicating complex health information. We developed AOM knowledge translation tools for Canadian parents and augmented them for Pakistani parent end users.

**Objective:**

This pilot study aimed to (1) develop AOM knowledge translation tools for Canadian parents, (2) adapt the knowledge translation tools across cultural contexts, and (3) evaluate the usability of the adapted knowledge translation tools.

**Methods:**

Parents’ perceptions of the translated knowledge translation tools’ usability were explored using a mixed-methods design. We recruited parent participants from a hospital in Pakistan to complete usability surveys (n=47) and focus group interviews (n=21). Descriptive statistics and content analysis were used to analyze data.

**Results:**

Usability results showed the usefulness and effectiveness of both adapted knowledge translation tools. Parents reported preferring a digital media narrative format in their own language. Findings revealed that culturally adapted knowledge translation tools are effective in transferring health information to parents.

**Conclusions:**

Digital knowledge translation tools offer a promising approach to improving health literacy and communicating complex health information to parents of children with AOM. Culturally adapting the tools generated important knowledge that will contribute to knowledge translation advancements. Evaluation of the tool effectiveness is a critical next step to exploring the impact of knowledge translation tools on child health outcomes.

## Introduction

Acute otitis media (AOM) is the most common pediatric bacterial infection, affecting up to 75% of children younger than 5 years [1,2]. AOM can cause pain in the ear, fever, and temporary hearing loss and is a leading cause of health care visits worldwide [2,3]. Despite the high incidence of AOM in children, it is often underrecognized and undertreated by clinicians [1]. Previous research identified that families want information about their child’s illness, expected treatments, and post–emergency department [4] or clinic care. However, the gap between evidenced-based research and end user knowledge remains large. Knowledge translation (KT) is increasingly recognized as a solution to bridge this gap.

KT is an iterative process of synthesizing, disseminating, and ethically applying knowledge to improve health, health services, and health care systems globally [5]. In child health settings, emphasizing parents’ role as partners in health care decision making reduces unnecessary health care use and ultimately improves health outcomes [5,6]. However, to be effective, parent health education should be multimodal and employ flexible, portable formats [7,8]. Incorporating illustrations and stories can also improve knowledge comprehension, retention, confidence, and compliance with discharge instructions [7-10]. Research has shown that innovative media (eg, digital and mobile technology, videos) are superior to traditional materials (eg, information sheets, pamphlets) in transferring information to consumers [8-14]. Previous research has illustrated the effectiveness of such tools in improving child health outcomes [8,11,13,15,16]. However, the scale-up of these digital KT tools for parents across different cultural contexts remains underdeveloped [16-18].

Closing the gap between research and practice has been consistently identified as a priority around the globe [19-21]. Despite this interest in KT, the best available research evidence is not consistently implemented in low- and middle-income countries (LMICs). Thus, the gap between research and practice are still increasingly wide in LMICs, where there are limited and scarce resources [21-24].

Digital or eHealth interventions have been identified as useful public health tools, particularly in underserved settings [25-29]. The availability and use of digital technologies, such as mobile phones, are increasing rapidly in LMICs [26,27]. eHealth interventions are useful in providing health information, reminders, emergency response, and monitoring [30]. In LMICs, digital interventions could reduce time, distance, and cost of information delivery, overcoming issues of inadequate financing, poor access to information, and limited human resources [31,32]. A wealth of research evidence is available from high-income countries on the effectiveness of digital health interventions. However, much less attention has been paid to how to augment these interventions to benefit LMICs.

The aim of this pilot project was to (1) develop digital KT tools on AOM for Canadian parents, (2) translate and augment the KT tools in a different cultural context, and (3) evaluate the usability of KT tools in a different cultural context (ie, Pakistani parents).

## Methods

### Overview

**Figure 1:**
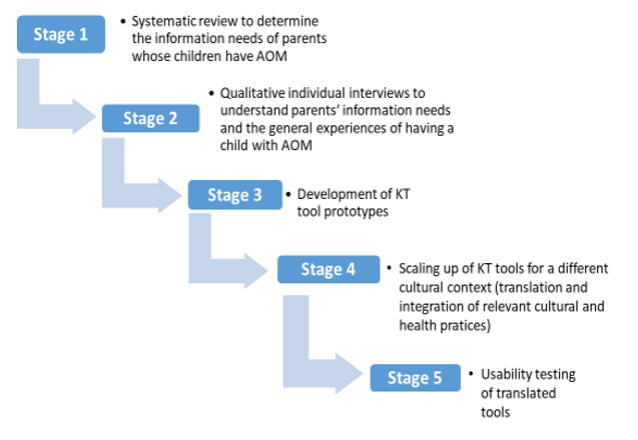
The 5-stage process of developing digital KT tools. AOM: acute otitis media; KT: knowledge translation.

This study used a person-centered approach for the design and development of digital arts-based KT tools, with mixed methods for usability evaluation. We developed the digital arts-based KT tools in 5 stages (Figure 1).

In stage 1, we conducted a systematic review to determine the information needs of parents whose children have AOM. The findings revealed that parents’ knowledge of AOM is generally limited. Further, parents were often poorly informed about AOM, resulting in uncertainties regarding how to help their children [33]. We conducted 16 individual qualitative interviews in stage 2 with parents who sought care for AOM in a hospital emergency department to understand their information needs and their experiences of having children with AOM [34]. Through thematic analysis, we found that AOM has considerable negative outcomes for both children and families (eg, pain, emotional strain for parents, etc) and that parents can benefit from evidence-based resources to meet their information needs [34].

The data generated from stages 1 and 2 were used to develop digital KT tools for Canadian parents. In stage 3, we developed KT tools (whiteboard video and infographic) for Canadian parents. After sharing the stage 1 and 2 results with a creative team (storywriter, graphic designer, and editor), we collaboratively developed KT tool prototypes. This involved creating a composite narrative (a compilation of common themes from the parental interviews), ensuring that the narrative integrated the best available research, developing artwork to complement the composite narrative, and creating a graphic display of the narrative and artwork. We shared the prototypes with clinicians and content experts to ensure the accuracy and appropriate interpretation of the evidence.

To expand the use of evidence-based interventions across cultural contexts, we augmented and translated the digital AOM KT tools. In stage 4, we revised the tools to integrate relevant cultural and health practices for Pakistani parent end users. Specifically, we accommodated for language, literacy level, educational background, and the availability of technology. Research from many LMICs, including Pakistan, revealed that weak pharmaceutical regulations have allowed people to access antibiotics as over-the-counter drugs [35], which leads to antibiotic resistance among children. Thus, cultural norms for antibiotic prescriptions for AOM, health care professionals’ roles in managing AOM, and parents’ roles in making health care decisions for their children were incorporated. The evidence on treatment for AOM remained the same; the only changes were the names of drugs for symptomatic management and an increased emphasis in the video and infographics on the proper usage of antibiotics for AOM. After the revision, a professional translator translated the tools into Urdu, the national language of Pakistan. Considering the average literacy level in Pakistan, we used grade 5–level Urdu to enable the majority of parents to understand the information. The first author, who is fluent in Urdu, rechecked the translated version to ensure its accuracy and appropriateness. Multimedia Appendices 1 and 2 show the English and translated infographics, entitled “How to Help When Your Child Gets an Ear Infection.” The whiteboard videos are available on YouTube in both English and Urdu (specially commended in the Institute of Human Development, Child and Youth Health Talks in 2018 and entitled “Mom! My ear hurts: What to do when your child has ear pain”) [36,37].

Before we disseminated these tools in Pakistan, in stage 5 we conducted a mixed-methods study (surveys and focus groups) to determine their usability, usefulness, and cultural appropriateness for Pakistani parents. The study received ethics approval from the University of Alberta’s Ethics Review Board. We also sought administrative approval from the medical director of the private hospital in Karachi, Pakistan, to recruit participants from that organization. We complied with all national regulations and laws that apply to foreign researchers for data collection in Pakistan.

### Usability Survey

To evaluate the usability of digital KT tools (whiteboard video and infographic), we used a 9-item 5-point Likert survey. The key elements included were informed by a systematic search of more than 180 usability evaluations, such as usability, aesthetics, language, level of engagement, quality of information, length, preference of form over traditional dissemination venues, and value added [38]. The survey was translated into Urdu and then back translated into English to verify its consistency and accuracy.

Between July 17 and August 4, 2017, we shared the augmented and translated digital tools with parents at a large private hospital in Karachi. Two local research assistants approached parents waiting at pediatric clinics. The inclusion criteria for study participation were (1) being a parent or guardian of a child aged 1 to 16 years, with past experience taking care of a child with AOM, (2) fluency in Urdu (speaking, hearing, reading, and writing), and (3) agreement from parents to be contacted by a research team for iPad usability testing. First, the research assistants explained the research purpose and process, and upon participant agreement, they completed demographic information forms. Next, an iPad was provided to participants to assess the translated KT tools. Completion and submission of the survey indicated parents’ consent. All of the participants viewed both tools in the same order, and the research assistants gave the paper-based usability survey forms to the parents. Parents also had an opportunity to provide free-text feedback on areas that required revisions or more information. The research assistants assisted parents who had difficulty understanding or filling out the survey forms. We cleaned and managed the data according to industry standards and entered them into IBM SPSS Statistics (version 23; IBM Corp) twice to ensure accuracy.

### Qualitative Focus Groups

We conducted 3 focus group interviews with parents to augment the survey findings with rich detail. Using purposive and convenience sampling, we recruited different participants from the same private hospital in which we previously administered usability surveys due to time limitations and the unavailability of previous survey participants. The inclusion criteria for study participation were (1) being a parent or guardian of a child aged 1 to 16 years, with previous experience caring for a child with AOM; (2) fluency in Urdu (speaking, hearing, reading, and writing); and (3) being a parent of children aged 1 to 16 years, being interested in participating in a focus group discussion, and not necessarily having experience caring for a child with AOM. After consenting to participate, the parents and guardians completed demographic information forms. The first author conducted 3 semistructured focus group interviews with 21 parents (5 to 9 parents per group). The interviews lasted from 45 minutes to 1 hour. At the beginning of each focus group, all participants viewed both tools. A semistructured interview guide was used to explore the participants’ perceptions of the translated KT tools (Multimedia Appendix 3). All the focus groups were conducted in Urdu, audiorecorded, transcribed verbatim, and translated into English by a professional translator. Data analysis via NVivo 11 (QSR International) used a conventional content analysis technique [39]. The first author read each transcript carefully and highlighted text including participants’ perceptions of the KT tools. All authors enhanced the analytic rigor by discussing the coding framework, analytic procedures, preliminary findings, and interpretations.

## Results

### Usability Survey Findings

We invited a total of 65 parents to participate in the usability survey, and 47 parents completed the survey forms. The majority (45/47, 96%) were mothers; 2 (4%) fathers participated. The ages ranged from 20 to 40 years (mean 30, SD 5.14 years). Table 1 shows the demographic characteristics of the study participants.

**Table 1:** Demographic characteristics of parents who participated in the usability testing survey and qualitative focus groups (N=68; 47 in usability survey and 21 in focus groups).

Variable		Participants, n (%)
**Gender**		
	Male	4 (6)
	Female	64 (94)
**Parent’s age (years)**		
	Younger than 20	1 (1)
	20-30	30 (44)
	31-40	29 (43)
	41-50	8 (12)
**Household income (RS)^a^**		
	Less than 15,000	6 (9)
	15,000-30,000	16 (24)
	31,000-60,000	20 (29)
	61,000-1000,000	14 (21)
	100,000 and above	5 (7)
	I do not want to share this information	7 (10)
**Highest level of education**		
	Some high school	12 (18)
	Some postsecondary	7 (10)
	Postsecondary certificate or diploma	3 (4)
	Postsecondary degree	4 (6)
	Graduate degree	35 (51)
	Other	7 (10)
**Total number of children in the house**		
	1	15 (22)
	2	23 (34)
	3	17 (25)
	4	10 (15)
	5	2 (3)
	6	1 (1)

^a^A currency exchange rate of RS 160.65=US $1 is applicable.

Overall, the majority of the participants (40/47, 85%) reported that both of the KT tools were useful and effective in communicating health information. All participants (n=47) strongly agreed or agreed that the tool instructions were very simple and easy to use. The majority of the parents (43/47, 91%) strongly agreed or agreed that these tools would help them make health care decisions for their children with AOM, and most reported that they would use the tools in the future and recommend them to their family members or friends (Table 2). The parents found these tools a great source of knowledge, stating that the tools raised their awareness of AOM. Additionally, participants reported that these tools would be useful for emergency cases.

**Table 2:** Frequency of participant answers on usability testing questionnaire (n=47).

Items	Strongly agree, n (%)	Agree, n (%)	Not sure, n (%)	Disagree, n (%)
The tools provide useful information	16 (34)	31 (66)	0 (0)	0 (0)
The tools provide information relevant to me	18 (38)	29 (62)	0 (0)	0 (0)
The tools are simple to use	17 (36)	28 (60)	2 (4)	0 (0)
I can use the tools without written instructions or additional help	13 (28)	19 (40)	7 (15)	8 (17)
Tools' lengths are appropriate	8 (17)	30 (64)	6 (13)	3 (6)
Tools are aesthetically pleasing	17 (36)	27 (58)	3 (6)	0 (0)
These tools help me to make decisions about my child's health	18 (38)	28 (60)	1 (2)	0 (0)
I would use these tools in the future	16 (34)	29 (62)	2 (4)	0 (0)
I would recommend these tools to a friend	20 (42)	24 (52)	3 (6)	0 (0)

### Qualitative Focus Group Findings

A total of 21 parents participated in the focus group discussion. Table 1 shows the demographic characteristics of the participants. We identified 3 major codes: (1) parents’ preference for KT tools (whiteboard video or infographics), (2) usability and feasibility of translated digital KT tools for parents, and (3) dissemination strategies.

#### Parents’ Preference for KT Tools

Parents’ reactions were generally positive. All parents in the focus groups preferred the whiteboard video to the infographic. They all considered the video more effective, as it provided details, including signs and symptoms and typical parental reactions. The parents also preferred the verbal information in the video, which made it possible to understand both audibly and visually. A few participants stated the infographic was useful, specifically in cases of limited technology access. A mother of a 4-year-old child stated:

Many people don’t have smartphones, so not necessarily all of them can watch video. If such [a] community is targeted,…it would be handier that they read this pamphlet.

#### Usability and Feasibility of Translated Digital KT Tools for Parents

All 21 focus group participants reported the information in both tools would be useful to families with young children. The majority agreed that the content was easy to understand. A mother of a 3-year-old child stated:

Information given in this video is in [a] very simple and easy [format] and in our own language [so] that everyone can comprehend it easily.

The participants also acknowledged that using the character-plot-narrative format humanizes health information, evokes emotional responses, and creates connection to the subject matter. A mother of 2 children younger than 6 years remarked:

I really like the story format; it’s really very interesting to see the parents, child, and doctor….Seems like I am seeing my personal experience.

The participants also discussed the importance of using digital media to communicate health information to parents. As a mother of 3 children younger than 8 years stated:

Such videos will be helpful for the mother, as she will be aware that these are the immediate remedial steps which she can take to help her child with ear pain.

Talking about Pakistani culture, one young mother said:

This kind of digital information is very beneficial for the girls who married at [an] early age. They don’t like that their mother or mother-in-law teach[es] them about their children’s healthcare. They believe more [in the] information available on social media or other digital platforms.

The participants also liked that the video was translated into Urdu and will be available on YouTube, making it more relevant to their culture. A father of 3 children said:

The tools in our own language provide an accurate account of what we feel as parents when our child experience[s an] ear infection.

They agreed that it is important to have health information in their local language.

#### Dissemination Strategies

The majority of parents preferred to have the KT tools disseminated through the private health organization’s established social media platform, such as YouTube or Facebook. One mother of 2 children younger than 6 years stated:

This private organization has a big name, and if you just make a blog page or a video interactive page where this video can be uploaded for easy access, a pop-up message will attract the website user to watch these videos as soon as anybody visits the website.

Some participants suggested that it would be helpful to show these kinds of videos in medical clinic waiting rooms to effectively use parents’ time. In addition, one female participant suggested a more direct dissemination method:

[The] hospital administration can make a broadcast list and send videos officially to parents. Hence, in the presence of a broadcast list, you can easily direct messages to parents, and [their] phone number is not shared [with] other persons,. . . so, the privacy will remain intact too.

## Discussion

### Principal Findings

Our findings demonstrate that culturally adapted translated KT tools permit parents’ receptivity to information, reassure them, and foster confidence. The parents found the tools usable and appreciated receiving digital health information in a narrative form in their own language. They considered these tools a great source of knowledge that raised their awareness on AOM. We found that the culturally adapted digital KT tools we developed have the potential to increase participant engagement and help parents in health decision making.

There is little evidence on how to best scale up digital KT interventions developed in Western countries to reduce child morbidity and mortality in LMICs. This study was an effort to address this gap. The usability evaluation revealed that modifying KT tools to fit a different language, culture, and lifestyle would potentially increase the evidence-based information to reach a greater number of parents.

Although culturally adapted digital KT interventions show great promise in improving child health outcomes, these interventions are rarely implemented. The assumption is that effective KT interventions in the specific context of a Western industrialized setting will not necessarily work in LMICs [40]. The World Bank argues that the scaling up of KT interventions should be “driven by a universalist process of simplifying rules and procedures for use by many people on a larger scale” [41]. With this in mind, we ensured that people with low literacy can easily understand the KT tools that we developed, and because digital and web-based technology is growing quickly in Pakistan, the scale-up process was straightforward [41].

Digital media can also facilitate the dissemination of evidence-based health care information without requiring significant amounts of health and human resources. Many LMICs are grappling with a crisis in human resources for health care caused by factors such as underinvestment in health and the brain drain of health professionals [42]. Coupled with the increased burden of disease and lack of affordable health care, the human resource crisis means that it is not practical to deliver services only through physical interactions. Hence, a growing number of practitioners are leveraging advances in communications technology to strengthen health care systems in Pakistan. The findings reveal that digital KT interventions have the potential to improve patient knowledge and are the preferred method to receive health information.

### Study Limitations

To the best of our knowledge, this is the first study conducted in an LMIC to evaluate the usability of culturally adapted digital KT tools. The findings of the study cannot be generalized to a broader population or other languages and cultures. Further, we recruited the sample from only one site, a large private hospital, so our sample potentially reflects parents who are educated and familiar with digital technology.

### Study Implications

Global Health 2035 made a powerful case for increasing investments in the development of new KT health tools and scaling up new and existing tools. This study provides valuable insights into scaling up digital KT tools for a different culture than they were originally intended for. Scaling up digital KT tools for use in different cultures can change the trajectory of child health globally. However, very little funding is available to many LMICs to conduct this type of research. In the United States, the National Institutes of Health and the Bill & Melinda Gates Foundation reported that 97% of research funding is directed to the development of new health technologies and only 3% to research into implementation [43]. Commenting on what they called the “3/97 gap,” the authors estimated that research into the development of new technologies could prevent about 22% of child deaths by scaling up the existing tools [44]. However, scaling up digital KT interventions and tools is not easy. Barriers include language and translation, literacy and education, culture, trust in Western interventions, cost of health care, public and private systems of care, use of unregulated private providers, unequal access to services, availability of digital technologies, and decision-making processes within families. Addressing these barriers and improving access to knowledge will improve the health outcomes. Our research team’s next step is to augment other digital KT tools to address common acute conditions in LMICs; develop guidelines for the adaptation of KT tools for different cultures, countries, and contexts; and evaluate the effectiveness of these digital tools in improving the health outcomes of children in LMICs.

### Conclusion

The process of scaling up digital KT tools discussed in this paper generated important new knowledge that contributes to the science of KT. On a global scale, several ongoing initiatives support scaling up successful digital health interventions. However, cultural adaptation in KT strategies and tools is critically important for the successful scale-up of digital health solutions. These novel findings highlight the potential for digital art-based KT tools, given their congruence with human communication and learning approaches. Our findings suggest that future research that involves digital art- and narrative-based tools for KT is needed and worthwhile; in particular, assessing these approaches with different types of clinical conditions (eg, acute vs chronic health conditions) and different types of parents (eg, demographics, educational levels, ethnic backgrounds) will be helpful. Future research to evaluate the acceptability of KT tools among local health care professionals and families as well as rigorous effectiveness evaluations of these tools are critical next steps to measuring the impact of KT tools on child health outcomes.

## Appendix

Multimedia Appendix 1 Infographic.

Multimedia Appendix 2 Infographic in Urdu.

Multimedia Appendix 3 Focus group interview guide.

## Abbreviations

AOM: acute otitis media
KT: knowledge translation
LMIC: low- and middle-income country

## References

[1] Qureishi A, Lee Y, Belfield K, Birchall J, Daniel M (2014). Update on otitis media - prevention and treatment. Infect Drug Resist.

[2] Venekamp R, Sanders S, Glasziou P, Del Mar CB, Rovers M (2015). Antibiotics for acute otitis media in children. Cochrane Database Syst Rev.

[3] Monasta L, Ronfani L, Marchetti F, Montico M, Vecchi Brumatti L, Bavcar A, Grasso D, Barbiero C, Tamburlini G (2012). Burden of disease caused by otitis media: systematic review and global estimates. PLoS One.

[4] Scott S, Albrecht L, Given L, Hartling L, Johnson D, Jabbour M, Klassen T (2018). Pediatric information seeking behaviour, information needs, and information preferences of health care professionals in general emergency departments: Results from the Translating Emergency Knowledge for Kids (TREKK) Needs Assessment. CJEM.

[5] (2014). Sources of Potentially Avoidable Emergency Department Visits. Canadian IFHI.

[6] Scott SD, Hartling L, O'Leary KA, Archibald M, Klassen TP (2012). Stories – a novel approach to transfer complex health information to parents: A qualitative study. Arts Health.

[7] Gittelman MA, Mahabee-Gittens EM, Gonzalez-del-Rey J (2004). Common medical terms defined by parents: are we speaking the same language?. Pediatr Emerg Care.

[8] Hartling L, Scott S, Pandya R, Johnson D, Bishop T, Klassen TP (2010). Storytelling as a communication tool for health consumers: development of an intervention for parents of children with croup. Stories to communicate health information. BMC Pediatr.

[9] Scott SD, Brett-MacLean P, Archibald M, Hartling L (2013). Protocol for a systematic review of the use of narrative storytelling and visual-arts-based approaches as knowledge translation tools in healthcare. Syst Rev.

[10] Archibald M, Scott S, Hartling L (2013). Mapping the waters: A scoping review of the use of visual arts in pediatric populations with health conditions. Arts Health.

[11] Graham ID, Tetroe J (2007). How to translate health research knowledge into effective healthcare action. Healthc Q.

[12] Boydell K, Gladstone B, Volpe T, Allemang B, Stasiulis E (2012). The production and dissemination of knowledge: A scoping review of arts-based health research. Forum Qualitative Sozialforschung.

[13] Hartling L, Scott SD, Johnson DW, Bishop T, Klassen TP (2013). A randomized controlled trial of storytelling as a communication tool. PLoS One.

[14] Houston TK, Allison JJ, Sussman M, Horn W, Holt CL, Trobaugh J, Salas M, Pisu M, Cuffee YL, Larkin D, Person SD, Barton B, Kiefe CI, Hullett S (2011). Culturally appropriate storytelling to improve blood pressure: a randomized trial. Ann Intern Med.

[15] Archibald MM (2016). Developing a Patient-Driven Arts-Based Knowledge Translation Tool for Parents of Children with Asthma [thesis]. University of Alberta.

[16] Aarons G, Sklar M, Mustanski B, Benbow N, Brown C (2017). "Scaling-out" evidence-based interventions to new populations or new health care delivery systems. Implement Sci.

[17] Milat A, Bauman A, Redman S (2015). Narrative review of models and success factors for scaling up public health interventions. Implement Sci.

[18] (2016). Scaling up projects and initiatives for better health: from concepts to practice. World Health Organization.

[19] Leach M, Tucker B (2018). Current understandings of the research-practice gap in nursing: A mixed-methods study. Collegian.

[20] Olswang LB, Prelock PA (2015). Bridging the Gap Between Research and Practice: Implementation Science. J Speech Lang Hear Res.

[21] (2017). mHealth: New horizons for health through mobile technologies. World Health Organization.

[22] Goyet S, Touch S, Ir P, SamAn S, Fassier T, Frutos R, Tarantola A, Barennes H (2015). Gaps between research and public health priorities in low income countries: evidence from a systematic literature review focused on Cambodia. Implement Sci.

[23] Lavis JN, Guindon GE, Cameron D, Boupha B, Dejman M, Osei EJA, Sadana R, Research to Policy and Practice Study Team (2010). Bridging the gaps between research, policy and practice in low- and middle-income countries: a survey of researchers. CMAJ.

[24] Lavis J (2009). How can we support the use of systematic reviews in policymaking?. PLoS Med.

[25] McCannon CJ, Berwick DM, Massoud MR (2007). The science of large-scale change in global health. JAMA.

[26] Agarwal S, Labrique A (2014). Newborn health on the line: the potential mHealth applications. JAMA.

[27] Chib A, van Velthoven MH, Car J (2015). mHealth adoption in low-resource environments: a review of the use of mobile healthcare in developing countries. J Health Commun.

[28] Clifford GD (2016). E-health in low to middle income countries. J Med Eng Technol.

[29] Qiang C, Yamamichi M, Hausman V, Miller R, Altman D (2012). Mobile Applications for the Health Sector. World Bank.

[30] Ormel H (2013). MHealth and eHealth, innovations in public health and SRHR: state of evidence, opportunities and challenges. Royal Tropical Institute Amsterdam.

[31] (2011). Amplifying the Impact: Examining the Intersection of Mobile Health and Mobile Finance: A discussion guide for collaborative insight presented by the World Economic Forum, in partnership with the Health Alliance. World Economic Forum.

[32] Labrique AB, Wadhwani C, Williams KA, Lamptey P, Hesp C, Luk R, Aerts A (2018). Best practices in scaling digital health in low and middle income countries. Global Health.

[33] Meherali S, Hartling L, Campbell A, Robin F, Scott S (2020). Parent information needs and experience regarding acute otitis media in children: A systematic review. Patient Educ Couns.

[34] Meherali S, Campbell A, Hartling L, Scott S (2019). Understanding Parents' Experiences and Information Needs on Pediatric Acute Otitis Media: A Qualitative Study. J Patient Exp.

[35] Khalid L, Mahsood N, Ali I (2016). The public health problem of OTC antibiotics in developing nations. Res Social Adm Pharm.

[36] Mom! My ear hurts: What to do when your child has ear pain. YouTube.

[37] Mom! My ear hurts: What to do when your child has ear pain. Translated Version. YouTube.

[38] Hornbæk K (2006). Current practice in measuring usability: Challenges to usability studies and research. Int J Hum Comput Stud.

[39] Hsieh HF, Shannon SE (2005). Three approaches to qualitative content analysis. Qual Health Res.

[40] Miranda JJ, Zaman MJ (2010). Exporting 'failure': why research from rich countries may not benefit the developing world. Rev Saude Publica.

[41] (2003). Scaling up the impact of good practices in rural development: a working paper to support implementation of the World Bank's rural development strategy. World Bank.

[42] Misau YA, Al-Sadat N, Gerei AB (2010). Brain-drain and health care delivery in developing countries. J Public Health Afr.

[43] Kuruvilla S, Schweitzer J, Bishai D, Chowdhury S, Caramani D, Frost L, Cortez R, Daelmans B, de Francisco A, Adam T, Cohen Robert, Alfonso Y Natalia, Franz-Vasdeki Jennifer, Saadat Seemeen, Pratt Beth Anne, Eugster Beatrice, Bandali Sarah, Venkatachalam Pritha, Hinton Rachael, Murray John, Arscott-Mills Sharon, Axelson Henrik, Maliqi Blerta, Sarker Intissar, Lakshminarayanan Rama, Jacobs Troy, Jack Susan, Jacks Susan, Mason Elizabeth, Ghaffar Abdul, Mays Nicholas, Presern Carole, Bustreo Flavia, Success Factors for Women’s and Children’s Health study groups (2014). Success factors for reducing maternal and child mortality. Bull World Health Organ.

[44] Kruk M, Yamey G, Angell S, Beith A, Cotlear D, Guanais F, Jacobs L, Saxenian H, Victora C, Goosby E (2016). Transforming Global Health by Improving the Science of Scale-Up. PLoS Biol.

