# Supplementary material for: Cultural Adaptation of Digital Knowledge Translation Tools for Acute Otitis Media in Low- to Middle-Income Countries: Mixed Methods Usability Study
Source: JMIR Form Res. 2021 Jan 20;5(1):e13908. doi: 10.2196/13908 (PMC7857946; doi:10.2196/13908)

How to Help When Your Child Gets an Ear Infection

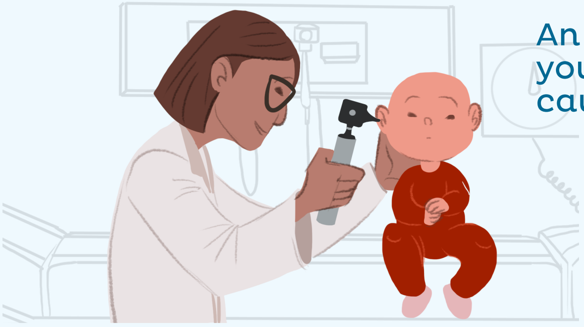

An ear infection, common in young children, is usually caused by a virus or bacteria.

Most infections clear up on their own in 2-3 days.

But they can be stressful for the whole family.

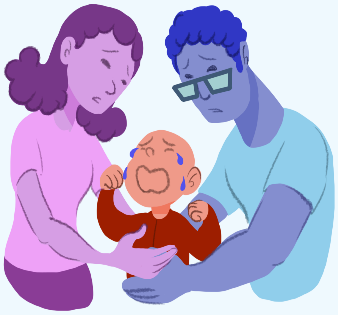

MILD SYMPTOMS

CHILDREN MAY:

- Have a mild fever (39˚C or less)
- Complain of ear pain
- Have difficulty sleeping or eating
- Cry and be "out of sorts"

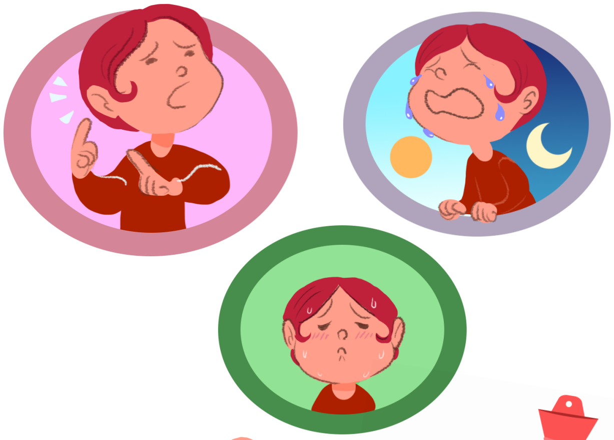

WHAT SHOULD YOU DO?

TIPS:

- Give your child children's Advil or Tylenol (for both pain and fever)
- Put extra pillows under your child's head
- Apply warm pack to ear

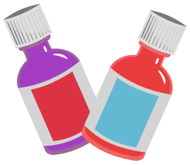

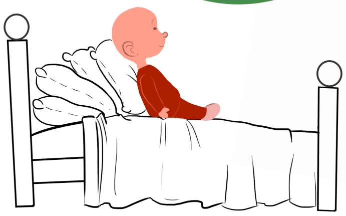

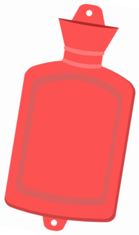

If your child does not get better after 48 hours, or has a fever above 39˚C, see a healthcare provider.

SEVERE SYMPTOMS

- Fluid coming out of the ear
- Seizures

WHAT SHOULD YOU DO?

If your child experiences SEVERE symptoms, go directly to an emergency department.

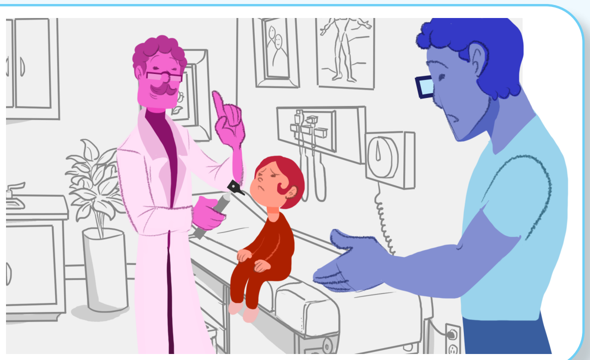

ANTIBIOTICS

A healthcare provider may prescribe antibiotics. Take antibiotics exactly as prescribed.

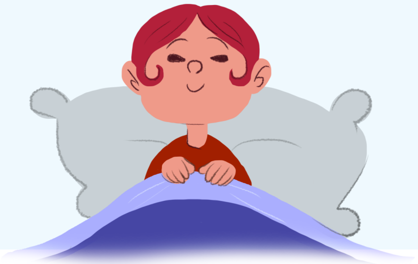

Supplement: Multimedia Appendix 1 [file formative_v5i1e13908_app1.pdf]
