# Supplementary figures and images for: Cultural Adaptation of Digital Knowledge Translation Tools for Acute Otitis Media in Low- to Middle-Income Countries: Mixed Methods Usability Study
Source: JMIR Form Res. 2021 Jan 20;5(1):e13908. doi: 10.2196/13908 (PMC7857946; doi:10.2196/13908)

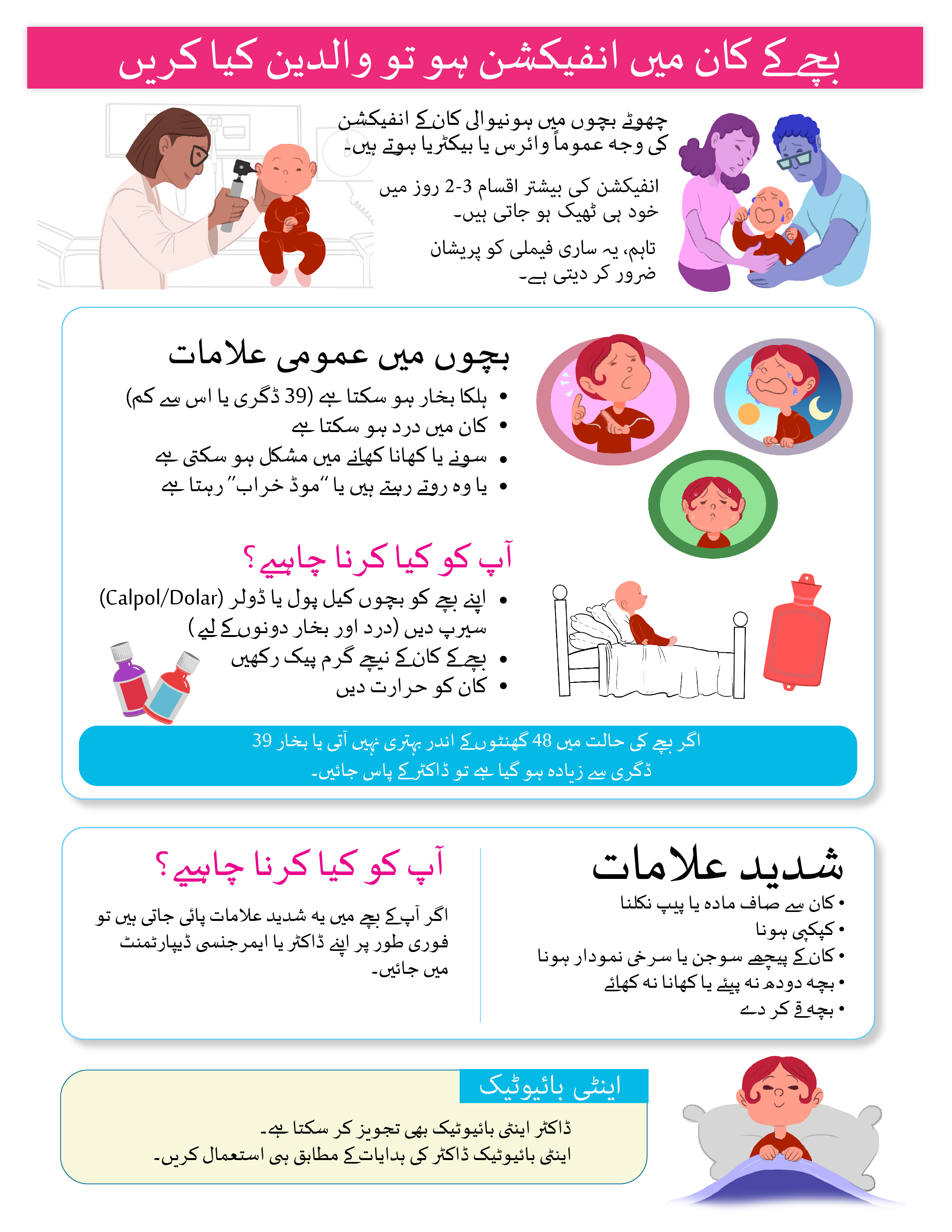

Supplement: Multimedia Appendix 2 [file formative_v5i1e13908_app2.png]
