# Supplementary material for: Cultural Adaptation of Digital Knowledge Translation Tools for Acute Otitis Media in Low- to Middle-Income Countries: Mixed Methods Usability Study
Source: JMIR Form Res. 2021 Jan 20;5(1):e13908. doi: 10.2196/13908 (PMC7857946; doi:10.2196/13908)
Supplement: Multimedia Appendix 3 [file formative_v5i1e13908_app3.docx]

**Focus Group Interview Guide**

Good morning/afternoon. Thank you for taking the time to meet with us. We would like to ask you several questions about your impressions of the tools. Our conversation is being transcribed to ensure that we have an accurate summary of your opinions. All the information we collect will be kept confidential. You may refuse to answer any questions or leave the focus group at any time. Do you have any questions before we begin? Please feel free to ask questions at any time during the interview.

Let’s get started;

1. Tell us which tool did you prefer?
2. Which tool did you like the least?
3. Tell me are there any tools that are tough or difficult to use or understand?
4. Do the tools accomplish the task of giving research information to parents and caregivers about their child’s health in a way that works for them?
5. What are your ideas on where we should put these tools so parents know about them?
6. Thinking of all the digital tools, how do they compare to the standard information sheet typically given by your hospital?
   1. What are some of the benefits and drawbacks of the digital tools vs the written information sheet?
   2. Which one would you prefer to find/receive:
      1. on the internet when you are looking for information about your child’s health?
      2. in a clinic/hospital waiting room?
      3. from health provider at the end of your clinic/hospital visit?
7. Your child has otitis media (ear infection). You're not sure what to do. You decide to search for information on the internet.
   1. What search terms (keywords) would you use in your search?
   2. Is there a particular website or place on the internet where you might look for information?
   3. What social media platforms do you use? Do you ever access health information on social media?

Thank you for your thoughtful responses to our questions. Are there any other comments/concerns about the digital tools that have not yet touched upon?
